# Pravastatin induces NO synthesis by enhancing microsomal arginine uptake in healthy and preeclamptic placentas

**DOI:** 10.1186/s12884-019-2507-0

**Published:** 2019-11-20

**Authors:** Zita Pánczél, Zoltán Kukor, Dorina Supák, Bence Kovács, András Kecskeméti, Rita Czizel, Magdolna Djurecz, Bálint Alasztics, Krisztián Benedek Csomó, András Hrabák, Sándor Valent

**Affiliations:** 1 2nd Department of Obstetrics and Gynecology, Semmelweis University, Üllői út 78/A, Budapest, 1088 Hungary; 2 Department of Medical Chemistry, Molecular Biology and Pathobiochemistry, Semmelweis University, Tűzoltó u. 37-47, Budapest, 1094 Hungary

**Keywords:** Preeclampsia, Placenta, Endothelial nitric oxide synthase, Pravastatin, arginine

## Abstract

**Background:**

Pravastatin, a known inducer of endothelial nitric-oxide synthase (eNOS) was demonstrated in human placenta, however the exact mechanism of it’s action is not fully understood. Since placental NO (nitric oxide) synthesis is of primary importance in the regulation of placental blood flow, we aimed to clarify the effects of pravastatin on healthy (*n* = 6) and preeclamptic (*n* = 6) placentas (Caucasian participants).

**Methods:**

The eNOS activity of human placental microsomes was determined by the conversion rate of C14 L-arginine into C14 L-citrulline with or without pravastatin and Geldanamycin. Phosphorylation of eNOS (Ser1177) was investigated by Western blot. Microsomal arginine uptake was measured by a rapid filtration method.

**Results:**

Pravastatin significantly increased total eNOS activity in healthy (28%, *p*<0.05) and preeclamptic placentas (32%, *p*<0.05) using 1 mM Ca^2+^ promoting the dissociation of a eNOS from it’s inhibitor caveolin. Pravastatin and Geldanamycin (Hsp90 inhibitor) cotreatment increased microsomal eNOS activity. Pravastatin treatment had no significant effects on Ser1177 phosphorylation of eNOS in either healthy or preeclamptic placentas. Pravastatin induced arginine uptake of placental microsomes in both healthy (38%, *p* < 0.05) and preeclamptic pregnancies (34%, *p* < 0.05).

**Conclusions:**

This study provides a novel mechanism of pravastatin action on placental NO metabolism. Pravastatin induces the placental microsomal arginine uptake leading to the rapid activation of eNOS independently of Ser1177 phosphorylation. These new findings may contribute to better understanding of preeclampsia and may also have a clinical relevance.

## Background

Preeclamsia is a gestational disorder characterized by hypertension and proteinuria, and is one of the leading cause of maternal and fetal mortality affecting 2–8% of all pregnancies [1, 2]. Since there have been no effective methods for its prevention and treatment, there is an urgent need to better understanding of the underlying mechanisms [3].

Preeclampsia is associated with widespread endothelial damage and decreased NO (nitric oxide) bioavailability. NO is produced by NO synthase from L-arginine, O_2_ and NADPH. Principally endothelial NO synthase (eNOS) isoenzyme is expressed by the human placenta [3], thus placenta has a crucial role in the development of preeclampsia.

The enzyme activity of eNOS is tightly regulated by several factors. eNOS activity is increased by elevated Ca^2+^ and tetrahydrobiopterin (BH4) levels, through its phosphorylation on Ser1177 by numerous kinases and dephosphorylation on Thr495 by phosphatases [3], and also by binding to the chaperone Hsp90. eNOS is activated by the bond of Hsp90. The Hsp90 inhibitors such as geldanamycin interact with the ATP binding site of Hsp90. Geldanamycin-bound Hsp90 resembles the ADP-binding conformation of the chaperone, and the replacement of ADP by ATP is not possible [4]. On the other hand, eNOS activity is decreased by the endogenous competitive inhibitor asymmetric dimethylarginine, through its dephosphorylation on Ser1177 and phosphorylation on Thr495 or by binding to caveolin-1, the scaffolding protein of caveolae [5, 6].

The activity of eNOS has been demonstrated to be also affected by pharmacological activators, statins. Statins are the known inhibitors of the rate-limiting enzyme of cholesterol biosynthesis, 3-hydroxy-3-methyl-3-glutaryl-Coenzyme A reductase [7]. Most recent statin therapies are based on their cholesterol lowering effects. They also have anti-diabetic, anti-inflammatory, antioxidant, neuroprotective, proangiogenic and anti-thrombotic properties, thus contributing to the endothelial protection. Moreover, statins were shown to decrease systolic and diastolic blood pressure in healthy and hypertonic participants [8, 9]. These protective effects of statins suggest that they have the therapeutic potential to treat preeclampsia.

Studies have shown that pravastatin can lower blood pressure and improve proteinuria in some preeclampsia-like rodent models (Nω-nitro-L-arginine methyl ester, inhibitor of eNOS and soluble vascular endothelial growth factor 1 receptor (sVEGFR-1)-induced mouse model) [10].

Statins affect eNOS activity in distinct ways, increasing eNOS expression by prolonging eNOS mRNA half-life. Simvastatin and lovastatin increase eNOS expression in human saphenous vein endothelial cell culture [11]. Statins modify the activity of eNOS by its phosphorylation on Ser615, Ser633 and Ser1177. Statins can activate the phosphatidylinositol 3-kinase (PI3K)/Akt pathway [12] and activate eNOS through the phosphorylation of Ser1177 and dephosphorylation on Thr495. Microsomes (subcellular fractions including endoplasmatic reticulum and cell membrane) have the highest activity of eNOS between placental subcellular fractions [13].

**Fig. 1:**
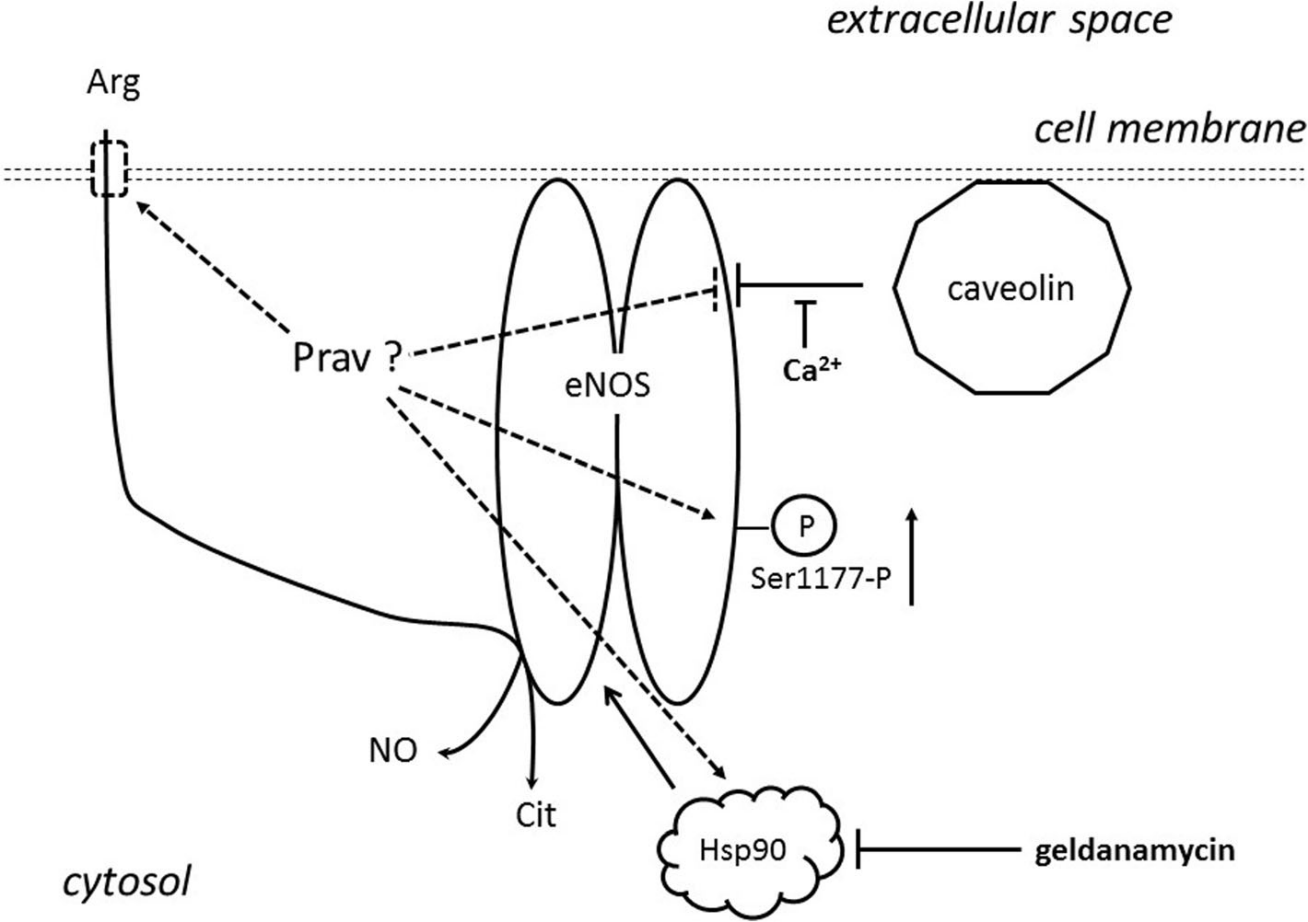
Potential effect sites of pravastatin on rapid eNOS activity

Figure 1 includes potential effect sites of pravastatin on rapid eNOS activity.

Recently, the first randomized pilot clinical study in humans has been published. In this study 20 high-risk patients (with a history of severe preeclampsia in a prior pregnancy) were treated with pravastatin versus placebo during pregnancy. While 40% of the placebo treated patients developed preeclampsia, patients that received pravastatin have not developed preeclampsia [14].

The teratogen effect of statin treatment under pregnancy is controversial. The risk of teratogenicity of statins appears to be relatively low according to a human study including women with hypercholesterolaemia (*n* = 64). Infants in the statin treated group had lower birth weight and shorter gestation period [15]. In a recent pilot clinical study, 40 mg pravastatin resolved the symptoms of preeclampsia of the patients. Blood pressure and urine protein/creatinine ratio of many participants had stabilized. Pravastatin was well tolerated, and there were no fetal or neonatal abnormalities at birth. Pravastatin reduced sVEGFR secretion from preeclamptic placenta in vitro [16].

## Methods

### Patients and human samples

Clinical data of the participants (control and preeclamptic pregnant patients) are shown as mean ± SD in Table 1 collected from medical records. Eligible preeclamptic caucasian race pregnant women have been diagnosed with preeclampsia according to the following criteria: presence of pregnancy-induced hypertension (systolic ≥140 mmHg, diastolic ≥90 mmHg) and proteinuria (≥300 mg/24 h or at least 2+) after the 20th week of gestation. Healthy caucasian race women with normal pregnancy were selected as controls, showing no signs of preeclampsia or other placental diseases. Participants were not treated with pravastatin during pregnancy. Written informed consent was signed by all participants to confirm their agreement to participate at the study. Human placentae were obtained randomly from 6 normal and 6 preeclamptic pregnancies at the 2nd Department of Obstetrics and Gynecology, Semmelweis University, Budapest, Hungary. All of our experiments were permitted by the Ethics Committee of the Medical Section of The Hungarian Academy of Sciences (Permisson No. 48995–2016/EKU). Placental tissue was immediately put into ice-cold transfer solution containing 0.9% NaCl, 40 mM HEPES/Na (pH = 7.4) and l mg/ml glucose and was transported immediately to the biochemical laboratory where the experiment was started without delay.

**Table 1:** Clinical data of participants

	Control pregnancies	Preeclamptic pregnancies
Maternal age (years)	29.0 ± 3.3	31.2 ± 2.3 n.s.
Delivery (weeks)	39.8 ± 0.4	33.5 ± 3.0^***^
Systolic blood pressure (Hgmm)	109.2 ± 8.6	163.3 ± 19.7^***^
Diastolic blood pressure (Hgmm)	74.0 ± 7.9	103.3 ± 8.2^***^
Proteinuria	0	++
Weight of fetus (g)	3435 ± 149	1993 ± 784^***^

*n.s.*not significant

^***^= *p*<0.001

#### Preparation of placental microsomes

The preparation of placental microsomes (MS) was carried out as previously reported [13]. Macroscopically isolated placental tissue mince was homogenized in 2 volume of ice-cold homogenizing solution containing 0.3 M sucrose, 40 mM HEPES/Na, pH = 7.4, 0.1 mM EDTA, 1 mM dithiothreitol, 1 mM phenylmethanesulfonyl fluoride, 10 μg/ml leupeptin, 10 μg/ml soybean trypsin inhibitor and 0.2 μg/ml aprotinin, using an UltraTurrax apparatus (IKA Werk, Staufen, Germany) at the three-quarter setting for 60 s. The homogenate produced by the UltraTurrax apparatus was filtered through a nylon mesh and cell debris and tissue fragments were removed by 2 min centrifugation at 600 g using a Janetzky K-24 refrigerated centrifuge. In order to separate cytosol and microsomal fractions, heavy particulate material and mitochondria were sedimented first at 15000 g for 30 min in a Beckman J2-HS centrifuge. The supernatant was then centrifuged at 100000 g for 60 min in a Beckman OptimaTM LE-80 K ultracentrifuge to obtain the microsomal pellet and the cytosol fraction. The pellet (MS fraction) was suspended in homogenizing solution. MS fraction was used immediatly to measure eNOS activity or refrigerated for further analysis at − 80 °C for western blot, or in liquid nitroge+ n for arginine uptake measurements.

#### Measurement of eNOS activity

Determination of NOS activity was performed by measuring the rate of conversion of radiolabelled L-arginine into radiolabelled L-citrulline as previously described [17, 18]. Control eNOS activities were determined without pravastatin. The eNOS activities were measured at 1 mM CaCl_2_, and 50 μM BH4 added in all experiments. In a typical experiment 3.5–5.5 mg MS protein was incubated.

#### Western blot analysis of eNOS phosphorylation

MS fraction was solved in sample buffer (2% sodium-dodecyl sulfat (SDS), 10 mM dithiothreitol), boiled for 5 min, and 50 μg protein was electrophoresed on 7.5% SDS–polyacrylamide gels. Electrophoresis was performed at 29 mA for 2 h using the Miniprotean II setup placed in ice-bath. Proteins were transferred to polyvinylidene difluoride (PVDF) membranes for 3 h at 290 mA, membranes were blocked overnight at 4 °C with TBST solution (20 mM Tris/HCl, pH 7.5, 100 mM NaCl, 0.1% Tween 20) containing 5% bovine serum albumine. Anti-eNOS was used a dilution of 1:1000 at room temperature for 2 h. Peroxidase-conjugated secondary antibody was used at 1:5000 dilution for 1 h at room temperature. Signals were developed by 1 min incubation with West-One chemiluminescent substrate and exposed to X-ray films for 0.5–5 min. Developed films were scanned using an Ultrascan XL Laser Densitometer.

#### Measurement of microsomal arginine uptake by rapid filtration method

Arginine uptake of placental MS were determinated by rapid filtration experiments. These determinations were essentially performed as described by Bánhegyi et al. [19] adapted to arginine uptake. MS (1 mg protein/ml) were incubated at 25 °C in microsomal homogenisating buffer (0.3 M sucrose, 40 mM HEPES/Na (pH = 7.4), 1 mM EDTA, 1 mM dithiotreitol, 1 mM PMSF, 10 μg/ml aprotinin, 10 μg/ml soytrypsin inhibitor, 5 μg/ml leupeptin) with or without 10 μM pravastatin for 10 min. This time 0.5 μCi C14 arginine has been added to the sample. At the indicated times 100 μl aliquots were withdrawn, filtered through cellulose acetate/nitrate filter membranes (pore size 0.22 μm), and quickly washed twice through the filter with 3 ml ice-cold homogenisating buffer containing 10 mM ornithine. The radioactivity retained on the filter was measured after the dissolvance of the filter by liquid scintillation. Alamethicin (50 μg/mg protein) has been included in parallel samples to distinguish the intravesicular radioactivities from the bound radioactivities. The alamethicin-releasable portion of radioactivity (assumed as intravesicular) was calculated by subtraction.

#### Protein determinations

Protein was measured by the method of Lowry et al. [20] using bovine serum albumin as standard.

#### Statistical analysis

Results were statistically evaluated by using Excel Data analysis softwares. The difference was accepted significant if *p* < 0.05. Test for statistically significant differences (p < 0.05) was performed by analysis of t-test. Data are given as mean ± SD for 3–6 separate experiments.

#### Materials

L-[U-14C] arginine (348 mCi/mmol; 12.9 GBq/mmol) were purchased from ICN (Costa Mesa, CA, USA). (6R)-5,6,7,8-tetrahydro-L-biopterin (BH4) was obtained from Research Biochemicals International (Natick, MA, USA). L-N^G^-nitroarginine methylester, NADPH, Dowex 50X8–400, dithiothreitol, L-citrulline, L-arginine, L-ornithine, calmodulin, leupeptin, alamethicin were from Sigma-Aldrich Kft. (Budapest, Hungary). Pravastatin, phenylmethylsulphonylfluoride and HEPES were from Calbiochem (La Jolla, CA, USA), and aprotinin from Bayer Co. (Leverkusen, Germany). Anti-eNOS, Anti-phospho-eNOS/NOS III (Ser1177) and donkey anti-rabbit IgG, peroxidase conjugated were obtained from Millipore (Boston, MA, USA). Other chemicals were from Reanal (Budapest, Hungary).

## Results

### Effect of pravastatin on eNOS activity

**Fig. 2:**
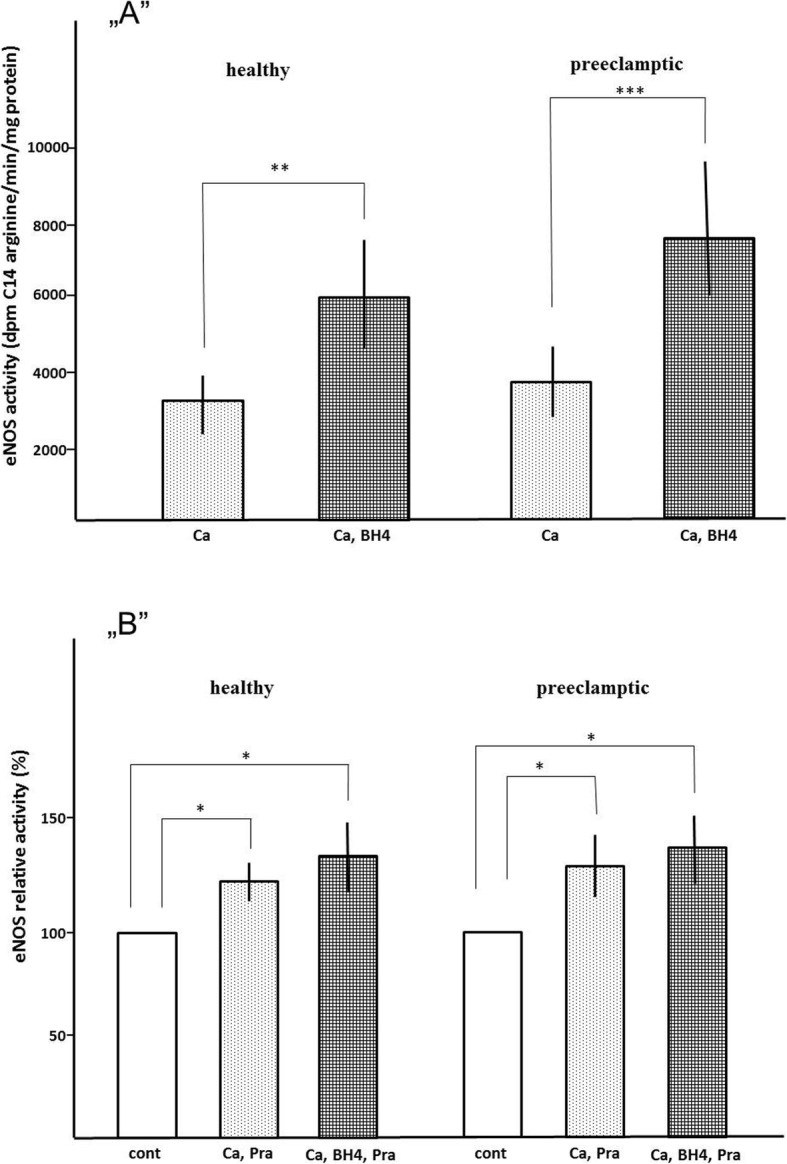
**,,A''** Microsomal eNOS activity. **,,B''** Effect of pravastatin on relative microsomal eNOS activity. These activities are the control data (100 %) of Figure 2. b. Activities are eNOS activity without pravastatin. **Ca**= 1 mM Ca^2+^; **Ca**, **BH4**= 1 mM Ca^2+^, 50 μM BH4. **= p<0.01, ***= p<0.001. Error bars show SD values (n=6). 100 % activities are eNOS activity without pravastatin. **Ca**, **Pra**= 1 mM Ca^2+^, 10 μM Pra; **Ca**, **BH4**, **Pra**= 1 mM Ca2+, 50 μM BH4, 10 μM pravastatin. *= p<0.05. Error bars show SD values (n=6)

Clinical data are summarized in Table 1. Consistent with our previous findings, we found that 50 μM BH4 enhanced eNOS activity approximately 2.0–2.5-fold in both groups (Fig. 2a). Figure 2b shows that 10 μM pravastatin treatment increased the maximal activity of eNOS (1 mM Ca^2+^, 50 μM BH4) in human placental MS both in healthy (28 ± 12%) and preeclamptic pregnancies (32 ± 10%). The difference was not significant (p = n.s.) between control and preeclamptic samples. The effect of pravastatin on eNOS activity proved to be dose dependent. The eNOS activities were increased appr. 30% by 10 μM pravastatin in 1 mM Ca^2+^, 50 μM BH4 (maximal activity). The activity of eNOS is decreased by 100 and 500 μM pravastatin in similar environments (data not shown). Caveolin inhibits eNOS in caveolae, but if Ca^2+^ levels are increased, caveolin dissociates from eNOS, and the enzyme is activated by 1 mM Ca^2+^.

### Pravastatin acts not only via phosphorylation of eNOS Ser1177

**Fig. 3:**
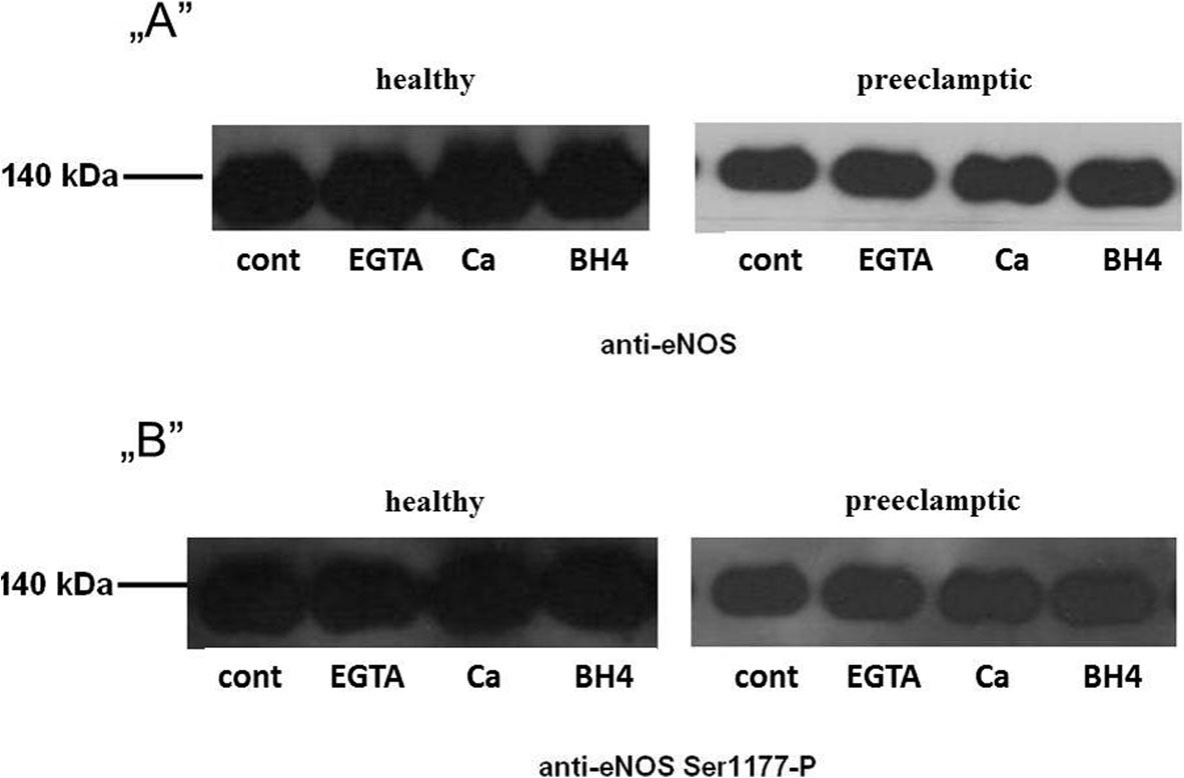
Effect of pravastatin on eNOS Ser1177. **„A”** is a typical result of western blot with anti eNOS antibody **„B”** is a typical result of western blot with anti eNOS phosphorylated on Ser1177 **cont** t= 0 min incubation, **EGTA**= 1 mM EGTA, 10 min incubation; Ca= 1 mM Ca^2+^, 10 min incubation and **BH4**=1mM Ca^2+^, 50 mM BH4, 10 min incubation

Pravastatin has pleiotropic effects, including phosphorylation of eNOS Ser1177. Samples were incubated under the same experimental condition (40 mM HEPES/Cl, pH = 7.4; 1 mM Mg^2+^; 1 mM NADPH; 1 mM dithiothreitol; 12 kU/L calmodulin; t = 10 min; T = 37 °C; EGTA, Ca^2+^, BH4 added as indicated) as used in measuring eNOS activity without radiolabeled L-arginine and investigating the phosphorylation of eNOS Ser1177 by western blot. We found that the phosphorylation status of microsomal eNOS Ser1177 did not change by 10 μM pravastatin (Fig. 3).

### Pravastatin action and Hsp90

**Fig. 4:**
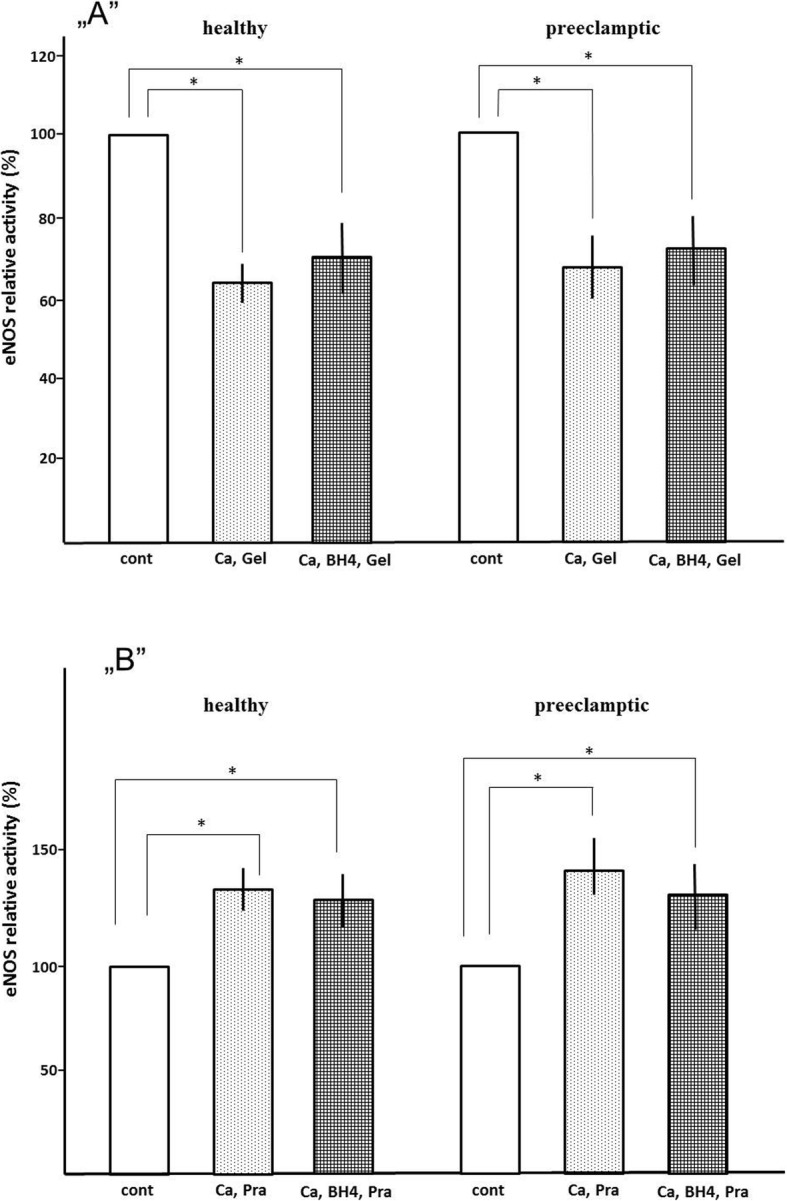
**„A”** Effect of 100 nM geldanamycin on eNOS relative activity of microsomes **„B”** The effect of pravastatin on relative eNOS activity of geldanamycin treatment microsomes. 100 % activities are eNOS activity without pravastatin and geldanamycin. **cont**= 1 mM Ca2+ or 1 mM Ca^2+^, 50 μM BH4; **Ca**, **Gel**= 1 mM Ca^2+^, 100 μM Geldanamycin; **Ca**, **BH4**, **Gel**= 1 mM Ca^2+^, 50 μM BH4, 100 μM Geldanamycin. *= p<0.05. Error bars show SD values (n=4)

ENOS is activated by the bond of Hsp90. The Hsp90 inhibitors such as geldanamycin interact with the ATP binding site of Hsp90. Geldanamycin-bound Hsp90 resembles the ADP-binding conformation of the chaperone, and the replacement of ADP by ATP is not possible [4]. The activity of eNOS is inhibited by 100 nM geldanamycin in both control and preeclamptic samples (Fig. 4a). Pravastatin (10 μM) moderately increases activity of eNOS (Fig. 4b). Geldanamycin (50 μM) attenuates NO production by eNOS in placental MS, while added pravastatin has no effect on eNOS activity.

### Effect of pravastatin on microsomal arginine uptake

**Fig. 5:**
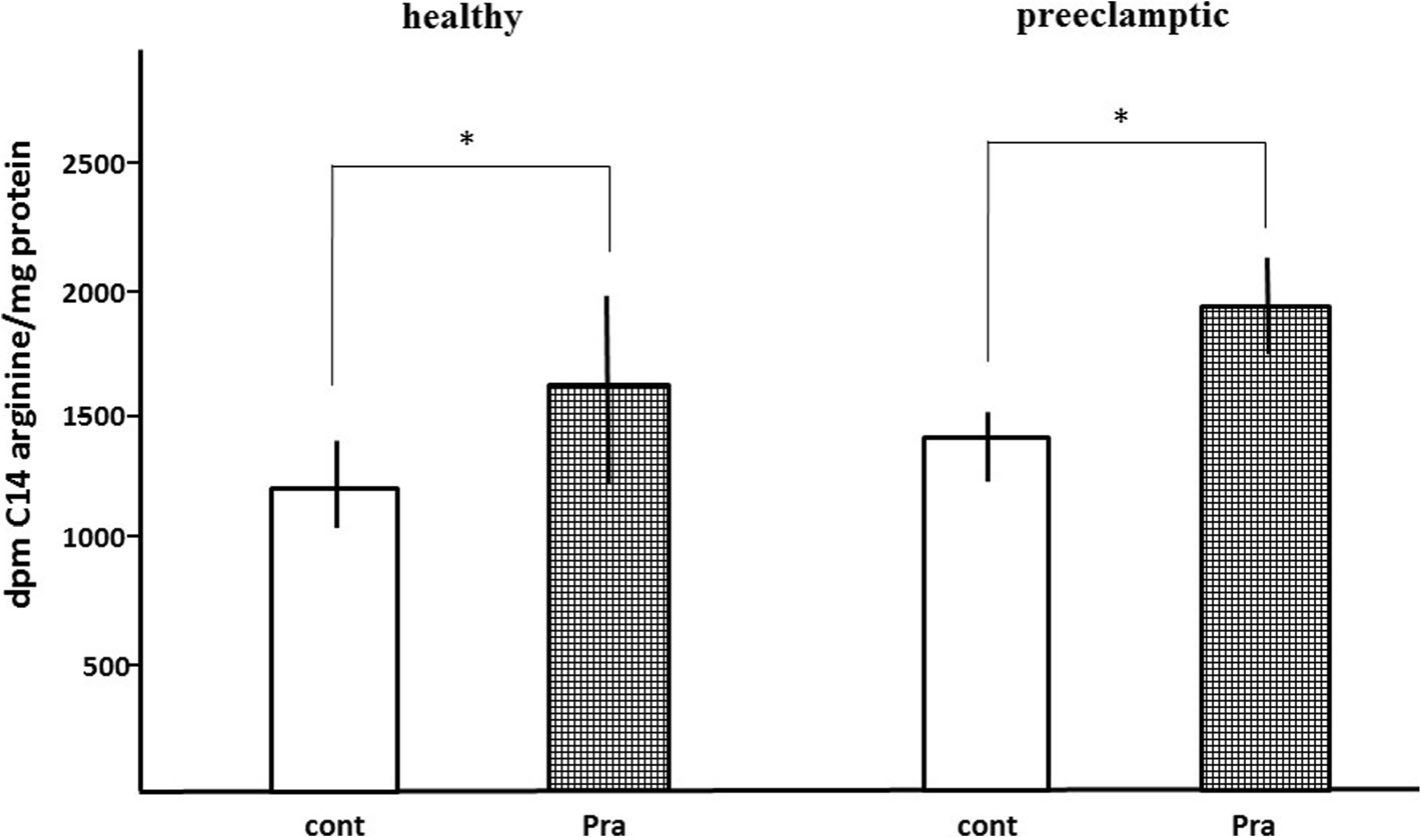
Arginine uptake of placental microsomes

The microsomal arginine uptake was time-dependent until the first minute. The arginine uptake was increased by 10 μM pravastatin in human placental MS both in healthy (38 ± 9%, *p* < 0.05) and preeclamptic pregnancies (34 ± 1%, *p* < 0.05). The difference was not significant (*p* > 0.05) between control and preeclamptic samples (Fig. 5).

## Discussion

Our recent experiments indicate that pravastatin can also increase eNOS activity rapidly. This effect is independent from the phosphorylation of eNOS Ser1177, caveolin, and Hsp90. According to our observations, activity of eNOS is increased by the rise of the enzyme’s substrate supply. Our findings suggest that pravastatin may improve arginine uptake of the placenta along with arginine substitution by insufficient arginine levels, thus decreasing the symptoms of preeclampsia. Our results of arginine uptake account for previous observations on the effect of statins.

Limitations of our study were: in vitro works, there are no preeclampsia samples grouped by severity (relatively small sample number), not classification of unknown arginine levels in caveola (localization site of arginine transporter and eNOS) of preeclamptic placentas. The average plasma L-arginine levels of patients with preeclampsia were significantly lower (54.2 μM) compared to healthy controls (85.4 μM) [21]. Homogenized primordal human placenta has 542 ± 142 μmol/kg wet tissue arginine concentration [22]. Arginine levels in preeclamptic villi were lower than arginine levels in homogenized villi from control, whereas arginine levels in the decidua were comparable in the two groups [23]. The difference between healthy and preeclamptic arginine concentrations may result in different eNOS activities and arginine transport.

The next step is to identify the arginine transporter that pravastatin affects.

Pravastatin stimulates human placental eNOS. Data suggests that pravastatin has both slow and rapid effects on eNOS activity [24–26]. Treatment of 100 nM pravastatin results in elevated expression levels of eNOS in human umbilical vein endothelial cell culture [27] and 200 nM pravastatin in cultures of placental explants [28]. The pravastatin induces PPARγ [29, 30] which results in decreased FFA levels, thus the inhibition effects of FFA on eNOS activity are reduced by pravastatin. FFA (arachidonic acid, palmitic acid, myristic acid and linolenic acid) decrease maximal eNOS activity in human placental MS during a short time (10 min.) incubation period (Z. Kukor’s and R. Czizel’s unpublished observations). According to data, reduced FFA levels may increase the activity of eNOS by distinct manners. Pravastatin has a short time effect on eNOS activity as well. Pravastatin can cause elevated NO production and vasorelaxation by phosphorylation on eNOS Ser1177 [31]. The stimulation of eNOS might be independent from the phosphorylation of eNOS Ser1177.We did not add exogeneous ATP (kinase subtrate) in our experiments. We found in our previous work that small molecules (arginine) were removed from MS by ultracentrifugation [22]. We suppose that MS still contains a small amount of its original ATP content, but 10 μM pravastatin does not cause phophorylation of eNOS Ser1177.

The presented results of arginine uptake may explain previous observations regarding to the effect of statins. Böger et al. investigated the effect of simvastatin on healthy volunteers’ endothelium-dependent vasodilation in the brachial artery. The effect of the combination of simvastatin and L-arginine on endothelium-dependent vasodilation was significantly greater than that of either simvastatin alone or L-arginine alone in the high serum level ADMA group. Simvastatin alone had no significant effect on endothelium-dependent vasodilation. Simvastatin, L-arginine, as well as their combination led to a significant increase in endothelium-dependent vasodilation in low serum level ADMA group [32]. ADMA is a competitive inhibitor of eNOS, thus these investigations are explicable by the potential effect of simvastatin on arginine uptake. Statin treatment increases the arginine-induced prolongation of the closure time of human platelets. The short time effect of statin can happen through the increase of eNOS activity, thus this effect can be contribute to the increase of arginine uptake [33].

Some contradicting results can be explained by the fact that pravastatin increases arginine uptake. By high arginine concentration (at least five times higher than the physiological concentration) pravastatin does not have a measurable effect on eNOS activity [28]. By low serum arginine concentration (ca. 50 μM) [21] or reduced arginine transport [34] pravastatin can increase arginine uptake significantly, thus increasing eNOS activity as well. It has been observed in preeclamptic pregnancies that arginine serum concentration is low [35].

Currently, statin treatment of pregnant women is contra-indicated. Theoretically, utilization of statins may help to fight against preeclampsia during pregnancy. The effect of pravastatin has been investigated in animal preeclampsia models by some recent studies. Ahmed A et al. reported that 20 μg/kg /day pravastatin treatment prevents preeclamptic features (induced hypertension, glomerular injury, increased sVEGF level, intrauterine retardation) in an immunologically-mediated preeclampsia mouse model that spontaneously develops pathological changes associated with preeclampsia [36]. Pravastatin restored angiogenic balance and prevented the appeariance of preeclamptic symptoms in CBA/J x DBA/2 mice. Insertion of adenoviral vector (AdV-) expressing sVEGFR into pregnant rats resulted in classic signs of preeclampsia. The low dose pravastatin treatment (5 μg/day) induced the expression of VEGF-like angiogenic factor placental growth factor and ameliorated the symptoms [37].

In some cases (e.g. in cases of pregnancies with higher risk) it is considerable to use (prava) statin treatment. A safe method could provide help in screening pregnant women with higher risk. A statin treatment may help reduce sVEGFR1 and FFA levels, which are usually increased in preeclampsia, and may also help handle disfunctions due to it.

## Conclusion

Our experiments indicate that pravastatin can also increase eNOS activity rapidly. This effect is independent from the phosphorylation of eNOS Ser1177, caveolin, and Hsp90. According to our observation, the activity of eNOS is observed to be increased by the rise of the enzyme’s substrate supply. Our findings suggest that pravastatin may improve arginine uptake of the placenta along with arginine substitution by insufficient arginine levels, thus decreasing the symptoms of preeclampsia. Our results of arginine uptake account for previous observations on the effect of statins. In our work, there was a difference in control (39.8 ± 0.4 weeks) and preeclamptic (33.5 ± 3.0 weeks) gestational age. We opinionate, that differences in gestational age could not have influenced the results. Expression of cationic amino acid transporters (CAT-1, 4F2hc and LAT-1) are not reduced in preeclamptic placenta (average gestational age 32.4 weeks of preeclamptic women and 37.6 weeks of healthy control women) [23].

## Supplementary information

**Additional file 1.** Detailed clinical data of participants.

## Data Availability

All data generated or analysed during this study are included in this published article (and its Additional file 1).

## Abbreviations

BH4: Tetrahydrobiopterin
EGTA: Ethyleneglycol-bis (2-aminoethylether) tetra-acetic acid
eNOS: Endothelial nitric oxide synthase
FFA: Free fatty acid
HMG-CoA3: Hydroxy-3-methyl-3-glutaryl-coenzyme A
Hsp90: Heat shock protein 90
MS: Microsomes
VEGF: Vascular endothelial growth factor
VEGFR: Vascular endothelial growth factor receptor
